# Stress Levels in Handball Coaching–Case Study: Preliminary Analysis of the Differences between Training and Match

**DOI:** 10.3390/ijerph191610251

**Published:** 2022-08-18

**Authors:** Nikola Foretić, Zoran Nikolovski, Dora Marić, Goran Gabrilo, Damir Sekulić, Damjan Jaksić, Patrik Drid

**Affiliations:** 1 Faculty of Kinesiology, University of Split, 21000 Split, Croatia; 2 Sport and Exercise Research Unit, Department of Psychological, Pedagogical and Education Sciences, University of Palermo, 90133 Palermo, Italy; 3 Faculty of Sport and Physical Education, University of Novi Sad, 21000 Novi Sad, Serbia

**Keywords:** anxiety, biomarkers, heart rate, trainer, STAI, saliva

## Abstract

Stress plays a significant role in competitions and in the training of sports participants, and coaches are no exception. To better cope with stressful situations, close monitoring of coaches’ stress levels before, during, and after training and competitions is recommended. According to studies, the use of cortisol (C) and alpha-amylase (AA) as biomarkers for monitoring acute stress is recommended. Therefore, the aim of our study was to compare HR, salivary C and AA, and STAI scores before, during, and after handball matches and training sessions. The study examined one professional handball coach, aged 37, in stress markers (salivary cortisol (C) and alpha-amylase (AA) concentrations), heart rate (HR), and the State-Trait Anxiety Inventory (STAI) scores in five matches/training sessions in the First Qatar Handball League. Statistical analysis included the calculation of descriptive statistic parameters, Mann–Whitney U test for differences between match–training time points, and the effect size analysis (Cohen’s d) to calculate the magnitude of differences between match–training time points. Presented markers (C and AA) had statistically stronger reactions before, during, and after the matches than the corresponding time points of the training sessions, similar to HR data and STAI scores. Results indicate that, before and during the matches, the analyzed markers of stress increased, which might lead to the conclusion that coaches are more anxious than frightened before and during matches. Thus, stress-coping strategies for handball coaches should be more focused on stress anticipation and anxiety control.

## 1. Introduction

Stress is a highly personalized phenomenon that varies between people depending on individual vulnerability and resilience and between different types of tasks [1]. Different people in different or similar situations experience stress differently. Stress is defined as a condition in which an individual is aroused and made anxious by an uncontrollable aversive challenge. It leads to a feeling of fear and/or anxiety [1]. An individual’s fight-or-flight response is triggered as a reaction to fearful situations. Stress is always connected to negative emotions (specifically, fear and/or anxiety), so its levels and physiological responses are influenced by a person’s perception of their ability to deal with the cause of stress. According to Kim and Diamond [2], stress has three major components: excitability/arousal, perceived aversiveness, and uncontrollability. Briefly, stress must increase neurochemical levels if a person perceives a situation as strongly aversive and uncontrollable.

Long-term exposure to stress, a characteristic of modern society, has a negative impact on overall health. Thotis [3] concluded that stressors, such as negative events, traumas, or strains, can have damaging effects on physical and mental health. Consequently, stress left unattended can affect organs and systems such as the nervous, gastrointestinal, cardiovascular, immune, and endocrine systems [4].

Different professionals are exposed to varying intensities of stress [5,6,7]. The literature shows that stress is mostly induced in professions and activities related to a competitive environment [8,9]. Sports competitions are no exception since competition induces significant stress in all participants [10]. Among them, coaches are exposed to specific competitive stress, and knowing how to deal with it has great importance and potential applicability in practice. Due to the nature of the coaching process, a sports coach can experience significant levels of stress. Hence, exploring the influence of stress on sports coaches is of interest to scientific inquiry and professionals who support and train athletes [11]. Coaches must play multiple roles and endure technical, physical, organizational, and psychological challenges in their activities. Therefore, it is not surprising that coaches experience stress as a result of the growing demands they encounter [12]. Those demands appear not only before, during, and after competitions but also before, during, and after sports training, which consumes most of a coach’s time and activities.

*Competitive stress* is defined as an ongoing transaction between an individual and the environmental demands directly associated with competitive performance. It is influenced by competitive stressors (environmental demands) and competitive strains (an individual’s negative psychological, physical, and behavioral responses to competitive stressors). In many cases, it results in competitive anxiety—a negative emotional response to competitive stressors [13].

In order to cope with stressful situations, close monitoring of the stress levels that coaches experience before, during, and after competitions and training is required. In the past, the main tools for monitoring were interviews or questionnaires [12,14]. This approach is adequate for measuring general stress but not sufficient for measuring acute stress occurring just before, during, and immediately after competitions and trainings [15]. A literature review shows that there is a lack of information concerning neurochemical responses to competition and training-related stress in sports coaches. Studies recommend the use of cortisol (C) and alpha-amylase (AA) as biomarkers for monitoring acute stress [16]. While the activation of the hypothalamus–pituitary–adrenocortical system (HPA) induces a substantial increase in cortisol levels and can be detected and measured in saliva, the sympathetic nervous system induces the release of alpha-amylase from salivary glands [17,18]. As these biomarkers can be collected non-invasively and fast, they are acceptable tools for measuring acute stress in sports coaches. Only two studies used C and AA as stress markers for coaches. Loupos et al. [19] studied salivary cortisol concentration in 8 coaches during a national swimming championship, while Hudson et al. [20] explored 10 male team sports coaches before, during, and after the match by assessing salivary AA activity. To the best of our knowledge, no study used both biomarkers simultaneously.

There are no studies that compare acute stress in handball coaches during matches and training. Hence, the main goal of this study was an investigation of a handball coach’s psychophysiological responses to competitions and training sessions by means of stress biomarkers (salivary C and AA, and HR) and an anxiety inventory (STAI). Specifically, the study aimed to compare salivary C and AA, HR, and STAI scores before, during, and after handball matches and training sessions. We hypothesized that handball coaches are differently exposed to stress during training and matches, and stress level is higher during matches than during training sessions. Accordingly, the rationale of the study was first to demonstrate the different levels of stress during matches and training sessions and, in further studies, propose, identify, and prove the slow existence of strategies to cope with stress.

## 2. Methods

### 2.1. Participants

One professional EHF master handball coach (kinesiology professor), with 19 years of coaching experience in handball, aged 37 (height 180 cm, weight 80 kg), participated in the study. During the course of the study, the subject coached a club in the First Qatar Handball League that was a cup finalist and at the end of the season was placed in 8th place.

### 2.2. Experimental Approach

**Scheme 1:**
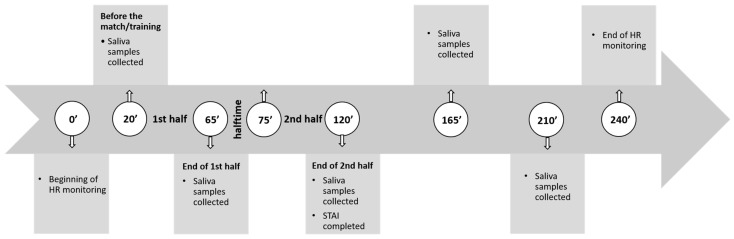
Data collection timeline, 20 min before the match/training, at halftime, directly after the match/training, and 45 and 90 min after the match/training.

Stress markers were measured during one rest day (baseline values), five training sessions, and five official matches (seven days from training) in the First Qatar Handball League. The coached team won three out of five games (1st game 34 to 29, 2nd game 30 to 34, 3rd game 32 to 28, 4th game 27 to 30, and 5th game 34 to 31). To establish the baseline circadian rhythm and stress biomarkers values (HR, C, and AA), the coach spent the day without physical activity, in his apartment fasting. In the course of the day, from 9:00 a.m. to 21:30 p.m., 10 saliva samples were taken, and HR was monitored at the same time points [21]. Baseline values corresponded to the same time of sample collection for training and matches, respectively. Due to the circadian variability of the measured markers, values varied during the day. Prior to collecting saliva samples, the subject stayed still (sitting) for 10 min, and during that period, HR was continuously monitored (the average values were taken as a result). In the second half of the study, stress markers were tracked before, during, and after the handball matches and training sessions (Scheme 1). All five matches and training sessions were held in the same hall (QHA sports hall), with 5000 seating places; the dimension of the court was 20 × 40 m, and the temperature ranged between 23 and 25 °C. The training sessions and matches were held in the same period of 35 days and were scheduled to take place between 16:30 p.m. and 20:30 p.m. Salivary cortisol (C) and alpha-amylase (AA) concentrations were measured, heart rate (HR) was monitored, and the State-Trait Anxiety Inventory (STAI) was completed.

### 2.3. Methodology

Saliva samples were taken at 5 time points: 20 min before the training/match, at halftime, directly after the training/match, and 45 and 90 min after the training/match (Scheme 1). The coach avoided eating a major meal an hour before sample collection and rinsed his mouth thoroughly with water 10 min before each sample was collected. For this purpose, SalivaBio Oral Swabs-SOS (Salimetrics LLC, State College, PA, USA) were used, placing them underneath the tongue on the floor of the mouth for 2 min. After collection, swabs were placed into a storage tube and immediately refrigerated. Within 2 h of sampling, samples were frozen at below −20 °C until centrifugation. On the day of analysis, samples were completely thawed and centrifuged at 1500× *g* for 15 min. After centrifugation, assays were performed. Saliva cortisol and alpha-amylase were analyzed with a commercially available enzyme-linked immunosorbent assay (ELISA) purchased from Salimetrics LLC (State College, PA, USA) on a microplate reader (Infinite 200PRO, Tecan, Mannendorf, Switzerland). All samples were analyzed in the same batch to avoid intra-assay variability.

Heart rate was measured using a heart rate monitor (Polar M430, Polar Electro, Kempele, Finland) that the coach wore for a total of 4 h: 30 min before the match/training, throughout the match/training, and 90 min after (Scheme 1).

The coach’s emotional state was determined with the State-Trait Anxiety Inventory (STAI) formY-1, which he completed himself after the match/training (Scheme 1). The STAI is an instrument that quantifies anxiety state and includes 20 questions that indicate how a participant felt at the moment they were completing the inventory. These questions are answered on the basis of a 1–4 scale, with the focus areas including worry, tension, apprehension, and nervousness. The result is presented as a score [22].

### 2.4. Statistical Analysis

The non-parametric/parametric nature of the variables was tested using the Kolmogorov–Smirnov test procedure. The calculation of descriptive statistic parameters included means, standard deviations, and percentages (for HR and biomarkers values). Furthermore, the Mann–Whitney U test was used to calculate the differences between training and match time points. The magnitude of differences between training and match time points was calculated by effect size analysis (Cohen’s *d*). Benchmarks used for interpretation of effect size were as follows: *d* = 0.2 was considered a small effect size, *d* = 0.5 a medium effect size, and *d* = 0.8 a large effect size. Statistica ver. 13.0 (Dell Inc., Austin, TX, USA) was used for the analyses, and a *p*-level of 95% (*p* < 0.05) was applied.

## 3. Results

**Figure 1:**
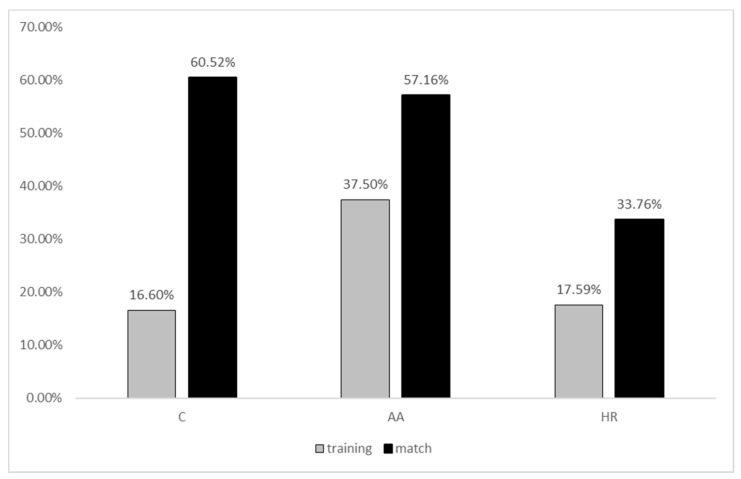
Pre-competitive effect: training and match increase compared with the baseline values presented as percentages (%).

Figure 1 shows that the biomarker values increased “before the training/match” time point compared with the baseline values. Data are presented as percentage increases for C, AA, and HR. There was a clear increase in all biomarker values before the training/match compared with baseline values. A greater increase was observed before the matches than before the training sessions across all biomarkers. The largest differences were for C, which before the match was 43.92% higher than before the training; AA was 19.66% higher, and HR was 16.17% higher.

Table 1 presents the results of the measured biomarkers for matches and training sessions. Differences were calculated using the Mann–Whitney U test. The magnitude of differences is presented as Cohen’s *d* (small, medium, and large). Significant differences were noted at particular time points before, during, and after the match/training, and the *p*-value was set at 0.05. Heart rate and C values showed a significant difference between matches and training sessions at all the time points of the analysis, except for the last one (90 min after the match/training). On the other hand, AA showed significant differences after the first and second half of the match/training and 90 min after the activity. However, the magnitude of differences measured by means of Cohen’s d showed a large difference across all time points and all biomarkers.

**Figure 2:**
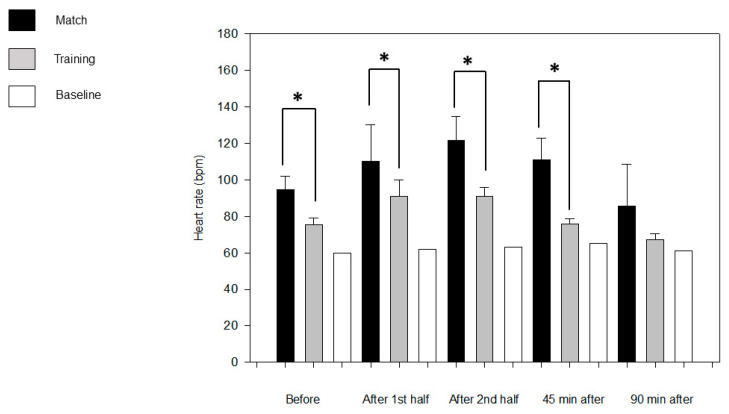
Heart rate (HR) values collected before, at halftime, directly after, and 45 and 90 min after, for the match, training, and the baseline with significant differences between match–training time points indicated with *.

Heart rate dynamics before, during, and after the match/training are presented in Figure 2. The results showed significantly higher values of HR throughout the matches than during training sessions at all time points except for the last one (90 min after the activity).

**Figure 3:**
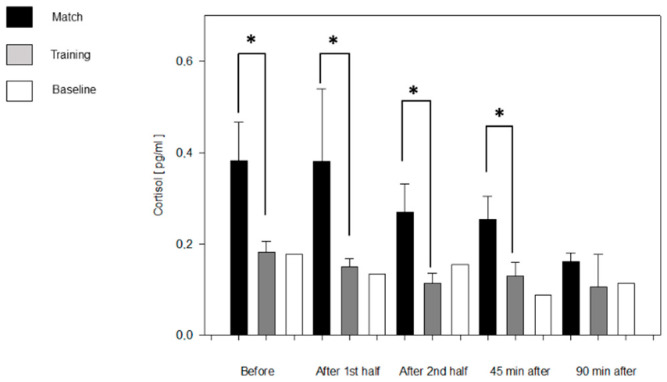
Salivary cortisol (C) values collected before, at halftime, directly after, and 45 and 90 min after, for the match, training, and the baseline with significant differences between match–training time points indicated with *.

Figure 3 shows the dynamics of cortisol and its differences before, during, and after the match/training. Similar to HR, C dynamics also showed significantly higher values during the matches than during training sessions, following the pattern of higher values throughout all time points except 90 min after the activity.

**Figure 4:**
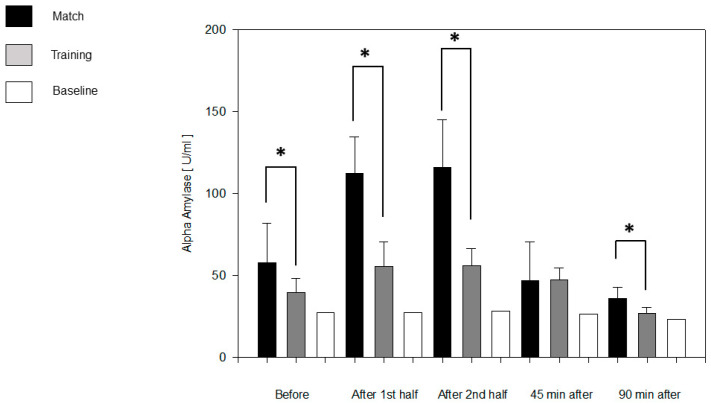
Salivary alpha-amylase (AA) values collected before, at halftime, directly after, and 45 and 90 min after, for the match, training, and the baseline with significant differences between match–training time points indicated with *.

Alpha-amylase dynamics and the differences in its levels relative to training and match time points are presented in Figure 4. As in all the previously presented markers, AA had a significantly stronger reaction before, during, and after the matches than the corresponding time points of the training sessions. Nonetheless, it is worth noting that even 90 min after the matches was an insufficient amount of time for AA to reach the baseline values, compared with 90 min after the training sessions.

**Figure 5:**
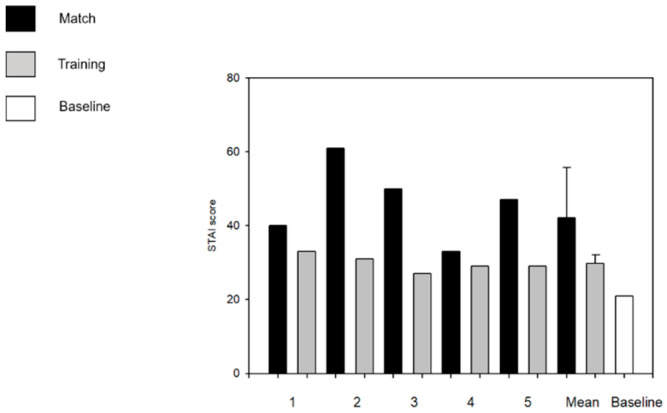
State-Trait Anxiety Inventory (STAI) values collected on five different matches, five different trainings, and at the baseline day.

The results of the STAI questionnaire scores after matches and training sessions are presented in Figure 5. Significantly higher scores were noted after the matches than after the training sessions. Obviously, the handball coach perceived the matches as more stressful than the training sessions. The highest score determined was 61 after a match and 33 after a training session. The difference between the mean STAI scores of the matches and training sessions was 14 points, which supports the previously mentioned observation that a match was more stressful than training from the coach’s perspective.

## 4. Discussion

To the best of our knowledge, this is the first study to compare the stress that a handball coach experiences during a match and during training. The study has three major findings: (1) There was an evident pre-competitive heightening of the coach’s emotional state; (2) for the coach, a handball match was more stressful than training; and (3) 90 min was a sufficient amount of time for HR and C to establish normal dynamics, while AA was still under the effect of the match stress. Accordingly, we may support our initial study hypothesis.

Before discussing the differences between training and match biomarker dynamics, it is important to mention the appearance of a “pre-competitive effect” that was noticed while comparing the biomarkers’ baseline and training/match values (Figure 1). This effect is attributed to either physiological arousal or psychological anxiety. Pre-competitive arousal and anxiety are well-documented phenomena related to a stronger psychological response to anticipating stressful situations [23,24,25,26]. A subjective appraisal of the ability to cope with competitive stressors suggests that they may develop negative emotional states and can disrupt decision making [26,27]. These negative emotions are expected to trigger a biological stress response through the activation of the sympathetic nervous system (observed as an increase in HR and AA) and the hypothalamic–pituitary–adrenal axis (observed as an increase in C) [28]. Preston et al. [26] found that speech anticipation stress provokes greater increases in heart rate in experimental subjects than in control participants. The anticipation of giving a public speech was effective as a stressor; it increased anxiety and heart rate only for participants in an anticipatory stress condition [26]. When it comes to handball coaches and their work, it is important to emphasize that they are exposed to many factors and situations challenging to control, especially before the match itself and also during training. Accordingly, it is logical that the coach expects these unpredictable circumstances, especially before a match, and such a state of anticipation causes anticipatory pre-competitive stress. Starcke et al. [29] reported similar findings, where anticipatory stress influenced not just HR but also AA and C response. In our study, all the measured biomarkers showed a pre-competitive increment, but it was most evident in C levels (Figure 1). Stronger C reactivity ahead of stressful situations is called anticipatory cortisol response [30]. According to van Paridon et al. [31], anticipatory cortisol response before a sports competition reflects moderate cortisol reactivity and optimally prepares athletes for the demands of sporting competitions via its influence on cognitive processes and attentional control. Since no study has dealt with this issue in sports coaches, we may speculate that coaches prepare for upcoming match challenges in a similar way to athletes. An emotional state of arousal is beneficial unless it induces anxiety [32]. The overall aim of a coach’s emotional state before the competition is to be aroused but not anxious. In such a state, a coach maximizes their performance [33]. In the context of handball coaches, it is important to mention that handball coaches must control 14 players and different game phases in socially complex situations/environments. More precisely, they need to control attacking and defending maneuvers and communicate with referees and colleagues (assistant coaches, doctors, physiotherapists, etc.) while keeping in mind the result of the game. In such a state, emotional stability is crucial and can only be achieved through optimal arousal without anxiety. The differences in stress response between training sessions and matches might originate from different sources. According to Frey [34], a coach’s stress emanates from interpersonal/personal factors (e.g., self-imposed expectations), influences of other people (e.g., athlete performance), task-related factors (e.g., decision making), and factors that would lead to them relinquishing their position (e.g., lack of enjoyment). The data from our study showed that differences in stress response are partly explained by the pre-competitive effect and additionally by the stress induced during match/training due to unexpected circumstances such as poor team performance, unexpected match/training dynamics, referee decisions, audience, and organizational issues [35,36]. When biomarkers were normalized with pre-competitive effect values, the data suggested that additional match/training stress was evident in AA and HR but not in C values (Figure 1, Figure 2, Figure 3 and Figure 4).

During matches and training sessions, a handball coach experiences different physical and emotional states. Throughout training sessions, a coach is more exposed to physical interventions (walking, hopping, jogging, passing the ball, shooting, etc.), while during a match (due to rules and restrictions), their physical interventions are very limited. There were significantly higher levels of HR, AA, and C during the matches compared with training sessions, even though training sessions normally last longer and are more physically demanding for coaches. These data suggested that the handball coach was more exposed to psychological stress during the matches. The STAI results corroborate this finding, where a large effect size was noticed between match and training STAI scores (Figure 5). Therefore, we may conclude that stress levels during training sessions do not resemble those during matches and that the reasons for higher stress during matches are unrelated to the physical demands of the match on the coach. Similar findings were reported by Hudson et al. [20], who examined stress responses in coaches during competitions, including psychological and physiological indices. Analyses comparing psychological responses between competition and non-competition days revealed significantly higher ratings on competition days in a number of stress-related variables: external/internal tension stress, external/internal effort stress, felt arousal, preferred arousal, and unpleasant emotions [20]. Accordingly, we may conclude that a handball coach is significantly more stressed during matches than during training sessions.

As expected, stress marker values declined after the matches and training sessions. The decline in HR, AA, and C after different kinds of physical activity has been reported in previous studies [36,37,38,39]. However, no study dealt with stress biomarker dynamics in sports coaches after stressful experiences. In order to better understand the dynamics of this decline, we measured marker dynamics at two separate points (45 and 90 min after the completion of the matches and training sessions). Comparing decreases in different stress biomarkers, we observed that AA continued to be elevated after the matches but not after the training sessions. On the other hand, C and HR returned to values close to those of the baseline as early as 90 min after both. Due to the intensity of the experience of coaching, a match might create a “*post-match effect*” in a coach. This kind of emotional state might create additional strain, where the coach undergoes a mental screening of immediately ended events. According to our results, additional strains were greater after the matches than after the training sessions, specifically considering AA values. The literature review showed a lack of studies that analyzed a coach’s stress levels after a competition. Hence, future research should focus on the amount of time needed for the normalization of stress biomarker levels, but also on possible stressors that might prolong increased stress biomarker levels once the competition is over.

### Limitations and Future Research Lines

The main limitation of this study is the small number of matches/training sessions used as the sample and the monitoring of only one coach. This makes the generalization of results limited since the study only focused on one subject. Future studies should include more coaches and a higher number of matches and training sessions.

## 5. Conclusions

For handball coaches, matches induce significantly higher stress than training. In our study, HR, C, and AA showed a significant increase before, during, and after the matches compared with those of the training sessions. The results of our study revealed the appearance of a “pre-competitive effect” observed as an increase in all the measured biomarkers before a match/training session began.

Differences in stress dynamics are mainly connected to a coach’s emotional state. Biomarkers show slightly different dynamics depending on stress system predominance (HPA or SNS). The data from this study suggested that, before and during matches, coaches a more anxious than frightened. Hence, stress-coping strategies for handball coaches should be more focused on anxiety control and stress anticipation. One of the techniques that could be conducted is *slow-paced breathing*, which consists of prolonged breathing exercises applied immediately before a match or training session. This technique might reduce a coach’s anxiety state. Future studies should corroborate or disprove such claims.

It is important to point out that a major part of a coach’s job is spent in training sessions. Bearing in mind the nature of handball as a sports game, training sessions should mimic the expected competitive stress. Although training seems less stressful than matches, a coach is also exposed to training stress. If this state continues for a prolonged period of time, it could provoke psychological burnout. Therefore, psychological preparation is needed for training, as it is for matches, and should not be neglected.

## Figures and Tables

**Table 1:** Differences between matches and training sessions; results calculated by Mann–Whitney U test and magnitude of differences presented as Cohen’s *d*.

Time Point	Heart Rate (bpm)		Cortisol (µg/dL)		Alpha-Amylase (U/mL)	
	Match X ± SD	Training X ± SD	Match X ± SD	Training X ± SD	Match X ± SD	Training X ± SD
**Before the match**	94.48 ± 7.60	75.24 ± 3.99	0.38 ± 0.08	0.18 ± 0.02	57.66 ± 24.07	39.52 ± 8.72
	*p* = 0.01 *; *d* = 3.29 ††		*p* = 0.01 *; *d* = 3.43 ††		*p* = 0.30; *d* = 1.12 ††	
**After 1st half**	110.04 ± 20.36	90.86 ± 8.89	0.38 ± 0.16	0.15 ± 0.02	112.37 ± 22.26	55.45 ± 14.99
	*p* = 0.05 *; *d* = 1.99 ††		*p* = 0.01 *; *d* = 2.30 ††		*p* = 0.01 *; *d* = 3.35 ††	
**After 2nd half**	121.56 ± 13.22	91.02 ± 5.00	0.27 ± 0.06	0.11 ± 0.02	116.27 ± 28.93	56.21 ± 10.51
	*p* = 0.01 *; *d* = 6.58 ††		*p* = 0.01 *; *d* = 3.58 ††		*p* = 0.01 *; *d* = 3.09 ††	
**45 min after the match**	111.04 ± 11.76	75.84 ± 2.94	0.25 ± 0.05	0.13 ± 0.03	46.94 ± 23.66	47.53 ± 7.19
	*p* = 0.01 *; *d* = 8.59 ††		*p* = 0.01 *; *d* = 2.91 ††		*p* = 0.83; *d* = 0.04	
**90 min after the match**	85.48 ± 22.95	67.22 ± 3.25	0.16 ± 0.02	0.10 ± 0.07	35.85 ± 7.15	26.99 ± 3.60
	*p* = 0.06; *d* = 1.39 ††		*p* = 0.14; *d* = 1.17 ††		*p* = 0.04 *; *d* = 1.75 ††	

Legend: *—significant differences at *p* < 0.05; ††—large magnitude of differences between match and training.

## Data Availability

The data presented in this study are available on request from the corresponding author. Data are not publicly available because they will be used in combination with other current research.
